# A familial Danish dementia rat shows impaired presynaptic and postsynaptic glutamatergic transmission

**DOI:** 10.1016/j.jbc.2021.101089

**Published:** 2021-08-18

**Authors:** Tao Yin, Wen Yao, Kelly A. Norris, Luciano D’Adamio

**Affiliations:** Department of Pharmacology, Physiology & Neuroscience, Brain Health Institute, New Jersey Medical School, Rutgers, The State University of New Jersey, Newark, New Jersey, USA

**Keywords:** familial Danish dementia, amyloid precursor protein, amyloid β, neurodegeneration, synaptic plasticity, rat, animal model, glutamate, BRI2, integral membrane protein 2B, Aβ, amyloid β, ACSF, artificial cerebrospinal fluid, AD, Alzheimer's disease, AMPAR, α-amino-3-hydroxy-5-methyl-4-isoxazolepropionic acid receptor, APP, amyloid-β precursor protein, FBD, familial British dementia, FDD, familial Danish dementia, imBRI2, immature BRI2, ISI, interstimulus interval, ITM2b, integral membrane protein 2B, KI, knock in, mBRI2, mature BRI2, mEPSC, miniature excitatory postsynaptic current, NFT, neurofibrillary tangle, NMDAR, *N*-methyl-d-aspartic acid, PPF, paired-pulse facilitation, Pr, probability of release, Rs, series resistance, SC, Schaeffer-collateral

## Abstract

Familial British dementia and familial Danish dementia are neurodegenerative disorders caused by mutations in the gene integral membrane protein 2B (*ITM2b*) encoding BRI2, which tunes excitatory synaptic transmission at both presynaptic and postsynaptic termini. In addition, BRI2 interacts with and modulates proteolytic processing of amyloid-β precursor protein (APP), whose mutations cause familial forms of Alzheimer's disease (AD) (familial AD). To study the pathogenic mechanisms triggered by the Danish mutation, we generated rats carrying the Danish mutation in the rat *Itm2b* gene (*Itm2b*^*D*^ rats). Given the BRI2/APP interaction and the widely accepted relevance of human amyloid β (Aβ), a proteolytic product of APP, to AD, *Itm2b*^*D*^ rats were engineered to express two humanized *App* alleles and produce human Aβ. Here, we studied young *Itm2b*^*D*^ rats to investigate early pathogenic changes in these diseases. We found that periadolescent *Itm2b*^*D*^ rats not only present subtle changes in human Aβ levels along with decreased spontaneous glutamate release and α-amino-3-hydroxy-5-methyl-4-isoxazolepropionic acid receptor–mediated responses but also had increased short-term synaptic facilitation in the hippocampal Schaeffer-collateral pathway. These alterations in excitatory interneuronal communication can impair learning and memory processes and were akin to those observed in adult mice producing rodent Aβ and carrying either the Danish or British mutations in the mouse *Itm2b* gene. Collectively, the data show that the pathogenic Danish mutation alters the physiological function of BRI2 at glutamatergic synapses across species and early in life. Future studies will determine whether this phenomenon represents an early pathogenic event in human dementia.

Model organisms that reproduce the pathogenesis of human diseases are useful to dissect disease mechanisms, identify therapeutic targets, and test therapeutic strategies. Because genetic manipulation has been easier in mice, mice have overtaken rats as the major rodent-based model organism in neurodegeneration research. Thus, to study familial Danish dementia (FDD) and familial British dementia (FBD), 15 years ago, we generated mice carrying the pathogenic Danish and British dementia mutations (integral membrane protein 2B [*ITM2b*]; *Itm2b*^*D*^ and *Itm2b*^*B*^ mice) into the *Itm2b* mouse gene (1, 2, 3). We choose a knock-in (KI) approach rather than the more common transgenic overexpression approach for several reasons. KIs mimic the genetic of FDD and FBD and make no assumption about pathogenic mechanisms (except the unbiased genetic one), whereas the transgenic approach aims to reproduce pathology (plaques, neurofibrillary tangles [NFTs], etc.), under the assumption that this “pathology” is pathogenic. In KI models, expression of mutant genes is controlled by endogenous regulatory elements, avoiding issues related to overexpression of disease proteins in a nonphysiological quantitative–spatial–temporal manner. Finally, potential confounding “insertion” effects of transgenes are avoided.

Because rats are better suited to study neurodegenerative diseases, we took advantage of recent developments in gene-editing technologies and introduced the familial Danish mutation into the genomic *Itm2b* rat locus (*Itm2b*^*D*^ rats). The rat was the organism of choice for most behavioral, memory, and cognitive research, which is critical when studying neurodegenerative diseases—because physiological processes are similar in rats and humans and the rat is an intelligent and quick learner (4, 5, 6, 7).

Several procedures that are important in dementia research are more easily performed in rats as compared with mice because of the larger size of the rat brain. Cannulas—to administer drugs, biologics, viruses, and others—and microdialysis probes—for sampling extracellular brain levels of neurotransmitters, amyloid β (Aβ), soluble tau, and others—can be accurately directed to individual brain regions, causing less damage and increasing specificity. *In vivo* brain imaging techniques, such as MRI (8) and PET (9, 10, 11), can assess the extent and course of neurodegeneration with better spatial resolution in rats. Moreover, rats are large enough for convenient *in vivo* electrophysiological recordings or serial sampling of cerebrospinal fluid for detection of biomarkers.

Finally, gene-expression differences suggest that rats may be advantageous model of neurodegenerative diseases over mice. For example, alternative spicing of *tau* (12, 13, 14, 15), which forms NFTs and is mutated in frontotemporal dementia (16, 17, 18, 19, 20, 21, 22, 23), leads to expression of tau isoforms with three or four microtubule-binding domains (3R and 4R, respectively). Adult human and rat brains express both 3R and 4R tau isoforms (24): in contrast, adult mouse brains express only 4R tau (25), suggesting that the rat may be a better model organism for dementias with tauopathy, such as FDD and FBD.

BRI2 physically interacts with and modulates processing of amyloid-β precursor protein (APP), which bears relevance to Alzheimer's disease (AD) pathogenesis (26, 27, 28, 29, 30). In addition, APP processing mediates long-term potentiation and memory deficits of Danish and British KI mice (31, 32, 33, 34, 35, 36). Aggregated forms of Aβ, a product of APP processing, are by and large considered the main pathogenic molecule in AD. Rat and human APP differ by three amino acids in the Aβ region: given that human Aβs are believed to have higher propensity to form toxic Aβ species as compared with rodent Aβs, we produced rats carrying the humanized Aβ sequence (*App*^*h*^ rats) (37, 38). Thus, to study possible interactions between the Danish mutation and human Aβ, *Itm2b*^*D*^ rats were backcrossed to *App*^*h*^ rats. Hence, all rats used in this study produce human and not rodent Aβ species.

Here, we studied periadolescent *Itm2b*^*D*^ rats, with the purpose of investigating early dysfunctions that may underlie initial pathogenic mechanisms leading to dementia later in life.

## Results

### Generation of *Itm2b*^*D*^ KI rats carrying humanized *App*^*h*^ alleles

The KI founder F0-*Itm2b*^*D*^ rat, which is carrying FDD mutation on *Itm2b* rat gene, was generated by CRISPR/Cas–mediated genome engineering as described in the Experimental procedures section and Supporting information. The F0-*Itm2b*^*D*^ rat, which is a chimera for the *Itm2b* gene, was crossed to WT (*Itm2b*^*w/w*^) Long–Evans rats to generate F1-*Itm2b*^*D/w*^ rats. F1-*Itm2b*^*D/w*^ rats were crossed to WT Long–Evans to generate F2-*Itm2b*^*D/w*^ rats. These crossings were repeated three more times to obtain F5-*Itm2b*^*D/w*^ rats. The probability that F5 rats carry unidentified off-target mutations (except those, if present, on chromosome 15) is ∼1.5625%. Male and female F5-*Itm2b*^*D/w*^ rats were crossed to obtain *Itm2b*^*D/w*^, *Itm2b*^*D/D*^, and *Itm2b*^*w/w*^ rats.

**Figure 1:**
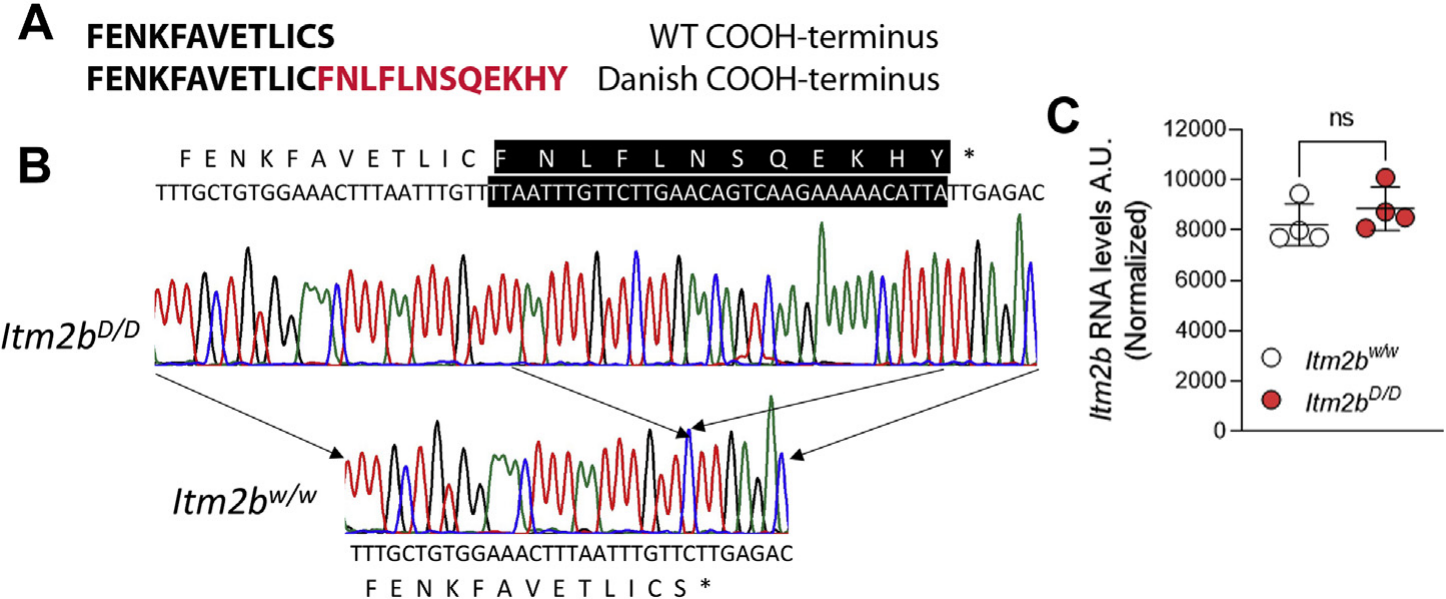
**Characterization of *Itm2b***^***D***^**KI rats.***A*, sequences of the COOH terminus of Bri2–23 (WT) and Bri2-ADan (Danish). *B*, PCR amplification and sequencing of the *Itm2b* gene exon 6 from *Itm2b*^*w/w*^ and *Itm2b*^*D/D*^ rats shows that the Danish mutation was correctly inserted in the *Itm2b* exon 6 of *Itm2b*^*D/D*^ rats. This mutation causes the predicted frameshift in the BRI2 sequence generating a precursor protein 11 amino acids larger-than-normal coding for the Bri2-ADan mutant protein (partial DNA sequences of WT and Danish exon 6 are shown). Inserted nucleotides are highlighted in *black*, and the amino-acid sequences are indicated above for the Danish mutant allele and below for the WT allele—the DNA sequences. *C*, levels of *Itm2b* mRNA in brains of 21-day-old *Itm2b*^*w/w*^ and *Itm2b*^*D/D*^ rats were determined by Standard-RNA-Seq analysis. No significant differences between *Itm2b*^*w/w*^ and *Itm2b*^*D/D*^ rats were evident. Data are represented as mean ± SD. Data were analyzed by Student's *t* test. N = 4 rats per genotype. KI, knock in.

The FDD mutation consists of a ten nucleotides duplication one codon before the normal stop codon (39). This produces a frameshift in the BRI2 sequence generating a precursor protein 11 amino acids larger than normal (Fig. 1*A*). To verify that the Danish mutation was correctly inserted into *Itm2b* exon 6, we amplified by PCR the *Itm2b* gene exon 6 *Itm2b*^*D/w*^, *Itm2b*^*D/D*^, and *Itm2b*^*w/w*^ rats. Sequencing of the PCR products shows that the Danish mutation was correctly inserted in the *Itm2b* gene exon 6 (Fig. 1*B*) and encoded for the COOH terminus of the Danish BRI2 mutant. When we generated FDD KI mice, we humanized the mouse COOH-terminal region of BRI2 by introducing an alanine (A) substituted for threonine (T) at codon 250 (3). Since that humanization did not result into deposition of ADan peptides in amyloid plaques in KI mice (3), that modification was not repeated in rats.

To generate *Itm2b*^*D/w*^, *Itm2b*^*D/D*^, and *Itm2b*^*w/w*^ rats on a background in which rat *App* has a humanized Aβ region, *Itm2b*^*D/w*^ and *App*^*h/h*^ rats were crossed to generate *Itm2b*^*D/w*^;*App*^*h/w*^ rats. The *App*^*w*^ allele was removed in subsequent crosses. Henceforth, *Itm2b*^*D/D*^, *Itm2b*^*D/w*^, and *Itm2b*^*w/w*^ rats used in this study have an *App*^*h/h*^ background and produce human and not rodent Aβ species.

To determine whether *Itm2b* expression is disrupted by the introduced mutations, we examined *Itm2b* mRNA levels in p21 *Itm2b*^*D/D*^ and *Itm2b*^*w/w*^ rats by standard RNA-Seq analysis on total brain RNA. The mRNA expression of *Itm2b* shows no significant difference between *Itm2b*^*D/D*^ and *Itm2b*^*w/w*^ rats (Fig. 1*C*).

### The *Itm2b*^*D*^ allele encodes for a longer Bri2 precursor protein (Bri2-ADan) that accumulates in primary neurons

BRI2 is type II membrane protein that is synthesized as an immature precursor (imBRI2). imBRI2 is cleaved at the COOH terminus by proprotein convertase to produce the NH_2_-terminal mature BRI2 (mBRI2) and the 23 amino acid–long COOH-terminal peptide called Bri23 (40). As noted previously, in the Danish patients, a frameshift caused by a ten nucleotides duplication 5′ to the stop codon leads to the synthesis of a BRI2 precursor protein 11 amino acids larger than normal (39). Convertase-mediated cleavage of Danish imBRI2 generates a WT-like mBRI2 and a 34 amino acid–long peptide called ADan, which codeposits with Aβ species in amyloid fibrils in patients. For clarity, we will refer to the WT imBri2 as Bri2–Bri23 and to the Danish mutant imBri2 as Bri2-ADan.

To determine whether the *Itm2b*^*D*^ allele codes for Bri2-ADan, we examined Bri2 expression in total neuronal lysates isolated from male and female 2-month-old *Itm2b*^*D/w*^, *Itm2b*^*D/D*^, and *Itm2b*^*w/w*^ rats. However, the Bri2 antibody tested identified many nonspecific bands (Fig. S1), making a rigorous assessment of Bri2 expression in rat brains challenging.

**Figure 2:**
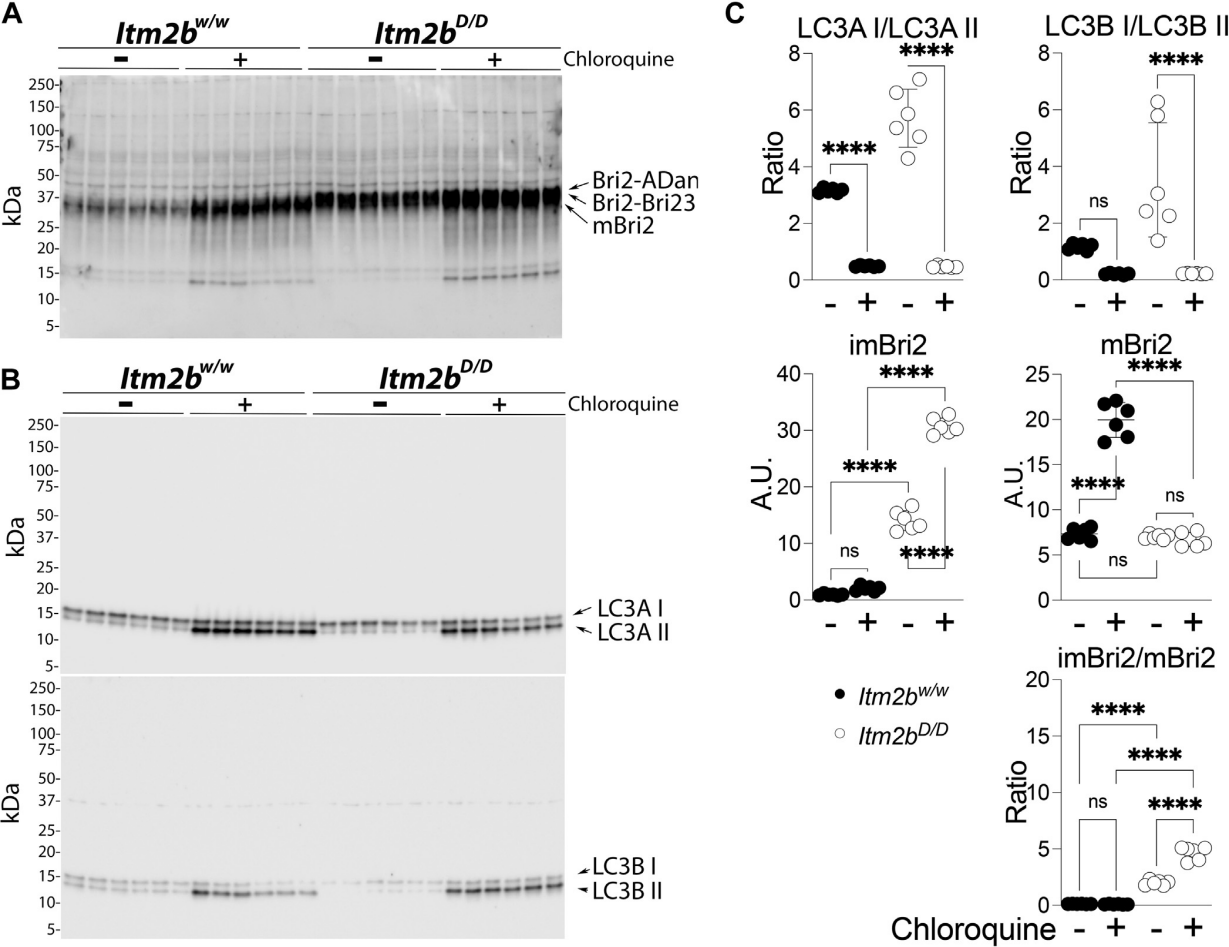
**Distinct degradation pathways of Bri2–23 and Bri2-ADan in *Itm2b***^***w/w***^**and *Itm2b***^***D/D***^**primary neurons.** WB analysis of Bri2 (*A*) and LC3A/B (*B*) from primary hippocampal neurons isolated from *Itm2b*^*w/w*^ (•) and *Itm2b*^*D/D*^ (○) P1 pups treated with either the vehicle PBS (−) or 50 μM chloroquine (+) for 18 h. *C*, quantification of LC3A/B and Bri2 levels. Data are represented as mean ± SD and analyzed by ordinary two-way ANOVA followed by post hoc Sidak's multiple comparisons test when ANOVA showed significant differences. Detailed statistical analysis results are shown in Table 1. WB, Western blotting.

Analysis of mouse *Itm2b*^*w/w*^ and *Itm2b*^*D/D*^ primary neurons showed that the mBri2/Bri2–Bri23 ratio in *Itm2b*^*w/w*^ primary neurons was significantly higher than the mBri2/Bri2-ADan ratio in *Itm2b*^*D/D*^ primary neurons (41). In addition, lysosomal inhibition caused accumulation of mBri2 but not Bri2–Bri23 in *Itm2b*^*w/w*^ primary neurons; in contrast, both mBri2 and Bri2-ADan accumulated in *Itm2b*^*D/D*^ primary neurons (41). These observations indicated that the Danish mutation reduced maturation of the mutant precursor Bri2 in mouse neurons. Based on these observations, we probed whether primary neurons could be used to assess mBri2, Bri2–Bri23, and Bri2-ADan expression in KI rats. Primary neurons are a simpler system compared with total brain; this, *per se*, may reduce the number of nonspecific bands identified by anti-Bri2 antibodies. Moreover, inhibition of lysosome-mediated degradation of Bri2 species in primary neurons may help identify specific Bri2 molecules. Thus, primary neurons derived from *Itm2b*^*w/w*^ and *Itm2b*^*D/D*^ rats were treated with the lysosomal inhibitor chloroquine and analyzed by Western blot. The anti-Bri2 antibody identified a band of ∼34 kDa in all samples, which was increased by chloroquine (Fig. 2, *A* and *C*). These observations are consistent with the ∼34 kDa corresponding to mBri2. A second band of ∼36 kDa was detected in *Itm2b*^*w/w*^ primary neurons (Fig. 2*A*). In contrast, a slightly larger second band (∼37 kDa) that was increased by chloroquine treatment was detected in *Itm2b*^*D/D*^ primary neurons (Fig. 2, *A* and *C*). These observations are consistent with the ∼36 and ∼37 kDa bands corresponding to Bri2–Bri23 and Bri2-ADan, respectively.

**Table 1:** Results of the statistical analysis of data shown in Figure 2

Ordinary two-way ANOVA analysis of Figure 2			
imBri2	Source of variation	F (DFn, DFd)	*p*
	Interaction	F (1, 20) = 272.2	<0.0001
	Treatment	F (1, 20) = 354.3	<0.0001
	Genotype	F (1, 20) = 1966	<0.0001
	Sidak's multiple comparisons test	Summary	Adjusted *p*
	*Itm2b*^*w/w*^ (Veh) *versus Itm2b*^*D/D*^ (Veh)	∗∗∗∗	<0.0001
	*Itm2b*^*w/w*^ (Veh) *versus Itm2b*^*w/w*^ (Chlo)	NS	0.5229
	*Itm2b*^*D/D*^ (Veh) *versus Itm2b*^*D/D*^ (Chlo)	∗∗∗∗	<0.0001
	*Itm2b*^*w/w*^ (Chlo) *versus Itm2b*^*D/D*^ (Chlo)	∗∗∗∗	<0.0001
mBri2	Source of variation	F (DFn, DFd)	*p*
	Interaction	F (1, 20) = 207.4	<0.0001
	Treatment	F (1, 20) = 186.9	<0.0001
	Genotype	F (1, 20) = 228.5	<0.0001
	Sidak's multiple comparisons test	Summary	Adjusted *p*
	*Itm2b*^*w/w*^ (Veh) *versus Itm2b*^*D/D*^ (Veh)	NS	0.9969
	*Itm2b*^*w/w*^ (Veh) *versus Itm2b*^*w/w*^ (Chlo)	∗∗∗∗	<0.0001
	*Itm2b*^*D/D*^ (Veh) *versus Itm2b*^*D/D*^ (Chlo)	NS	0.9966
	*Itm2b*^*w/w*^ (Chlo) *versus Itm2b*^*D/D*^ (Chlo)	∗∗∗∗	<0.0001
imBri2/mBri2	Source of variation	F (DFn, DFd)	*p*
	Interaction	F (1, 20) = 108.3	<0.0001
	Treatment	F (1, 20) = 104.2	<0.0001
	Genotype	F (1, 20) = 627.1	<0.0001
	Sidak's multiple comparisons test	Summary	Adjusted *p*
	*Itm2b*^*w/w*^ (Veh) *versus Itm2b*^*D/D*^ (Veh)	∗∗∗∗	<0.0001
	*Itm2b*^*w/w*^ (Veh) *versus Itm2b*^*w/w*^ (Chlo)	NS	>0.9999
	*Itm2b*^*D/D*^ (Veh) *versus Itm2b*^*D/D*^ (Chlo)	∗∗∗∗	<0.0001
	*Itm2b*^*w/w*^ (Chlo) *versus Itm2b*^*D/D*^ (Chlo)	∗∗∗∗	<0.0001
LC3AI/II	Source of variation	F (DFn, DFd)	*p*
	Interaction	F (1, 20) = 37.36	<0.0001
	Treatment	F (1, 20) = 353.5	<0.0001
	Genotype	F (1, 20) = 36.16	<0.0001
	Sidak's multiple comparisons test	Summary	Adjusted *p*
	*Itm2b*^*w/w*^ (Veh) *versus Itm2b*^*w/w*^ (Chlo)	∗∗∗∗	<0.0001
	*Itm2b*^*D/D*^ (Veh) *versus Itm2b*^*D/D*^ (Chlo)	∗∗∗∗	<0.0001
LC3B I/II	Source of variation	F (DFn, DFd)	*p*
	Interaction	F (1, 20) = 8.111	0.0099
	Treatment	F (1, 20) = 26.75	<0.0001
	Genotype	F (1, 20) = 8.283	0.0093
	Sidak's multiple comparisons test	Summary	Adjusted *p*
	*Itm2b*^*w/w*^ (Veh) *versus Itm2b*^*w/w*^ (Chlo)	NS	0.2185
	*Itm2b*^*D/D*^ (Veh) *versus Itm2b*^*D/D*^ (Chlo)	∗∗∗∗	<0.0001

Abbreviation: NS, not significant.

∗∗∗∗ indicates *P* < 0.0001.

**Table 2:** Results of the statistical analysis of data shown in Figure 3*C*

Ordinary one-way ANOVA analysis of Figure 3*C*		
Protein	F (DFn, DFd)	*p*
sAPPα	F (2, 27) = 0.1084	0.8977
sAPPβ	F (2, 27) = 0.7666	0.4744
Aβ38	F (2, 27) = 0.1121	0.8943
Aβ40	F (2, 27) = 2.030	0.1509
Aβ42	F (2, 27) = 4.764	0.0169∗
Aβ43	F (2, 26) = 2.654	0.0893
Aβ42/Aβ40	F (2, 27) = 4.074	0.0284∗
Aβ43/Aβ40	F (2, 26) = 4.031	0.0299∗
Aβ43/Aβ42	F (2, 23) = 3.281	0.0558


	Tukey's multiple comparisons test	Summary	Adjusted *p*
Aβ42	*Itm2b*^*w/w*^*versus Itm2b*^*D/w*^	NS	0.6966
	*Itm2b*^*w/w*^*versus Itm2b*^*D/D*^	∗	0.0159
	*Itm2b*^*D/w*^*versus Itm2b*^*D/D*^	NS	0.0948
Aβ42/Aβ40	*Itm2b*^*w/w*^*versus Itm2b*^*D/w*^	NS	0.8326
	*Itm2b*^*w/w*^*versus Itm2b*^*D/D*^	∗	0.0301

Abbreviation: NS, not significant.

∗ indicates *P* < 0.05.

Without treatment, the levels of Bri2-ADan in *Itm2b*^*D/D*^ primary neurons were significantly higher than the levels of Bri2–Bri23 in *Itm2b*^*w/w*^ primary neurons (Fig. 2, *A* and *C*), and the mBri2/Bri2–Bri23 ratio in *Itm2b*^*w/w*^ primary neurons was significantly higher than the mBri2/Bri2-ADan ratio in *Itm2b*^*D/D*^ primary neurons (Fig. 2, *A* and *C*).

LC3A and LC3B are autophagosome membrane proteins: the LC3A I/LC3A II and LC3B I/LC3B II ratios for each provides an independent measure of the impact of chloroquine on lysosomal degradation. Chloroquine significantly reduced the LC3A I/LC3A II and LC3B I/LC3B II ratios (Fig. 2, *B* and *C*), confirming inhibition of lysosome-mediated degradation.

### Subtle increase in Aβ42 levels in young *Itm2b*^*D*^ KI rats

Sequential processing of APP by α-/γ-secretase and β-/γ-secretase generates the following APP-derived peptides and polypeptides: sAPPβ, sAPPα, β-CTF, α-CTF, AID/AICD, P3, and Aβ. Since BRI2 interacts with APP and modulates APP processing by α-, β-, and γ-secretase (26, 27, 28, 29, 30), we determined the steady-state levels of several of these APP metabolites in the central nervous system of young male and female *Itm2b*^*D*^ KI rats. Full-length APP, α-CTF, and β-CTF were measured by Western blot: soluble APPs (sAPPα/sAPPβ) were detected by ELISA, and human Aβ species (Aβ38, Aβ40, Aβ42, and Aβ43) were detected by human Aβ-specific ELISA. These measurements have previously been used for other KI rats generated in our laboratory (38, 42, 43).

**Figure 3:**
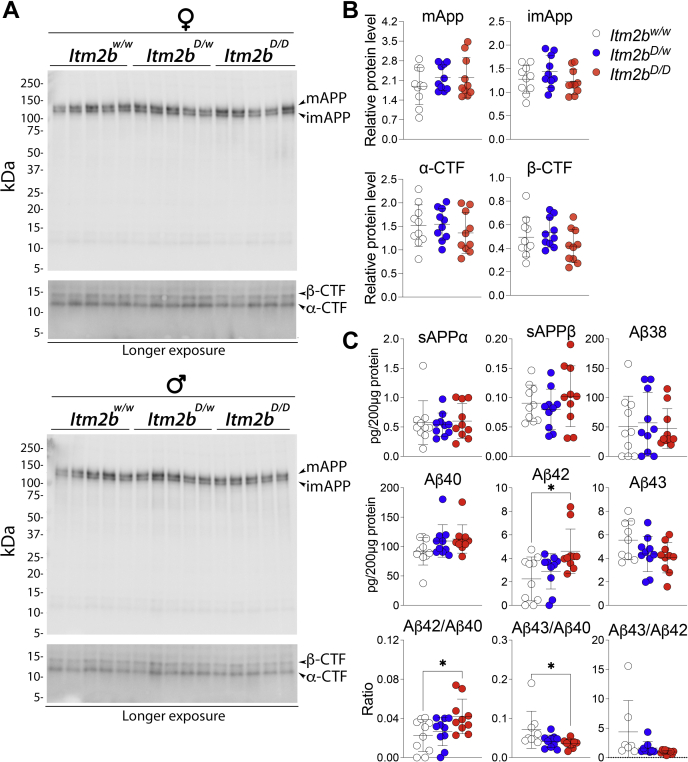
**Levels of peptides and polypeptides derived from APP processing in *Itm2b***^***D***^**KI rats.** Data are represented as mean ± SD and were analyzed by ordinary one-way ANOVA followed by post hoc Tukey's multiple comparisons test when ANOVA showed statistically significant differences. We analyzed 8-week-old rats and five female and five male rats per genotype. *A*, levels of full-length APP, αCTF, and βCTF were determined by Western analysis of brain lysate of *Itm2b*^*D/D*^, *Itm2b*^*D/w*^, and *Itm2b*^*w/w*^ male and female rats. *B*, quantitation of Western blots. Signal intensity of APP metabolites were normalized to red Ponceau staining of nitrocellulose membranes. Data were analyzed by ordinary one-way ANOVA. ANOVA summary: mAPP, F_(2, 27)_ = 0.7931, *p* = 0.4627; imAPP, F_(2, 27)_ = 1.367, *p* = 0.2720; α-CTF, F_(2, 27)_ = 0.6075, *p* = 0.5520; β-CTF, F_(2, 27)_ = 1.614, *p* = 0.2177. *C*, levels of sAPPα, sAPPβ, Aβ38, Aβ40, Aβ42, and Aβ43 were determined by ELISA of brain lysate from the same *Itm2b*^*D/D*^, *Itm2b*^*D/w*^, and *Itm2b*^*w/w*^ male and female rats. Data were analyzed by ordinary one-way ANOVA. Detailed statistical analysis results are shown in Table 2. APP, amyloid-β precursor protein; KI, knock in.

Neither the levels of full-length APP, CTFs, Aβ38, Aβ40, Aβ43, sAPPα, and sAPPβ were unchanged in 8-week-old *Itm2b*^*D/w*^, *Itm2b*^*D/D*^, and *Itm2b*^*w/w*^ rats (Fig. 3, *A*–*C*) nor was the Aβ43/Aβ42 ratio altered (Fig. 3*C*). In contrast, there was a slight but significant increase in Aβ42 as well as the Aβ42/Aβ40 ratio in *Itm2b*^*D/D*^ compared with *Itm2b*^*w/w*^ rats (Fig. 3*C*). Small but statistically significant decreases in both Aβ43 and Aβ43/Aβ42 ratios were evident in *Itm2b*^*D/D*^ as compared with *Itm2b*^*w/w*^ rats (Fig. 3*C*). Overall, these data indicate a gene dosage–dependent minor increase in steady-state levels of Aβ42, and decrease in Aβ43, in periadolescent *Itm2b*^*D*^ rats. Analysis of older rats will be needed to determine whether the Danish mutation in *Itm2b* alters APP processing in KI rats and whether these alterations may more robustly change the steady-state levels of APP metabolites with aging.

**Figure 4:**
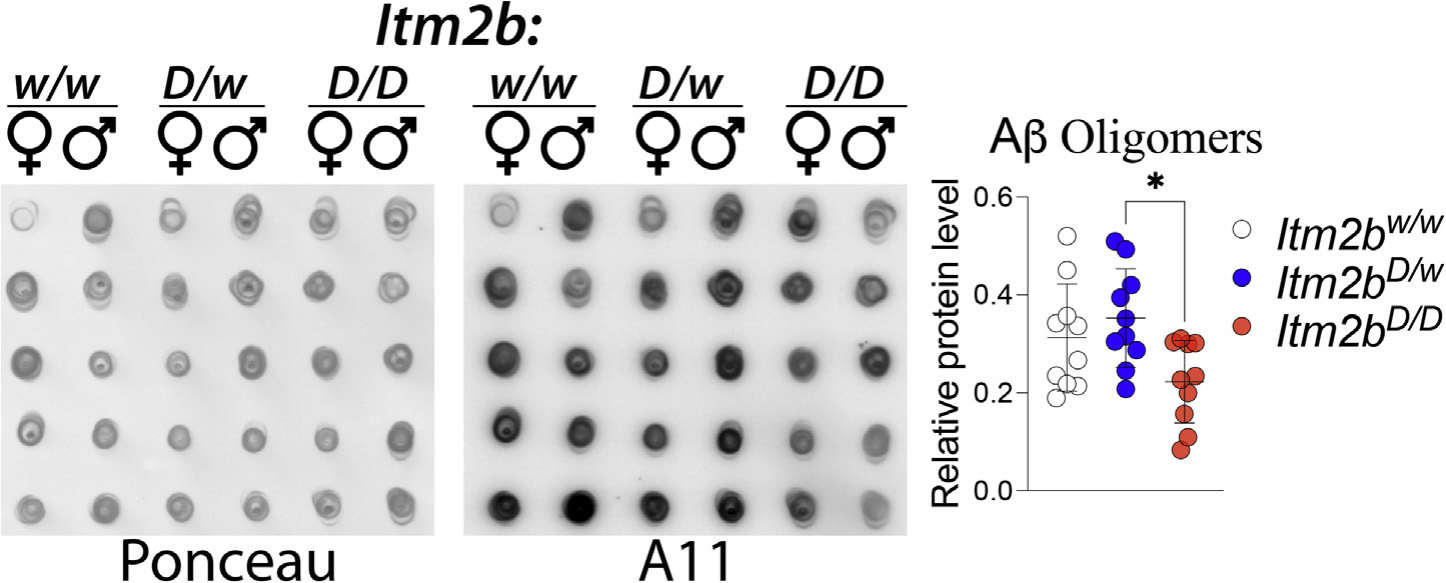
**Levels of human Aβ oligomeric species in the brain of periadolescent *Itm2b***^***D/D***^**, *Itm2b***^***D/w***^**, and *Itm2b***^***w/w***^**male and female rats.** We analyzed material from the same rats analyzed in Figure 3. Quantitation of dot blots using the oligomer-specific antibody A11. Before immunoblot analysis, membranes were stained with Ponceau red. Quantitative analysis of A11 blot was normalized to the Ponceau red quantitative analysis. Data are represented as mean ± SD and analyzed by ordinary one-way ANOVA followed by post hoc Tukey's multiple comparisons test when ANOVA showed statistically significant differences. ANOVA summary: F_(2, 27)_ = 4.593, *p* = 0.0192∗; post hoc Tukey's multiple comparisons test: *Itm2b*^*w/w*^*versus Itm2b*^*D/w*^, *p* = 0.6406, *Itm2b*^*w/w*^*versus Itm2b*^*D/D*^, *p* = 0.1195; *Itm2b*^*D/w*^*versus Itm2b*^*D/D*^, *p* = 0.0170∗.

It has been postulated that toxic forms of Aβ are oligomeric (44). Thus, we tested whether toxic oligomers are augmented in periadolescent *Itm2b*^*D*^ rats. To this end, we used the prefibrillar oligomer-specific antibody A11 to perform dot blots (45). We found no evidence supporting an increase in neurotoxic brain oligomer levels in periadolescent *Itm2b*^*D/w*^ and *Itm2b*^*D/D*^ rats as compared with *Itm2b*^*w/w*^ rats (Fig. 4). However, Aβ oligomers appeared to be significantly increased in *Itm2b*^*D/w*^ rats compared with *Itm2b*^*D/D*^ animals (Fig. 4). Analysis of older rats will be needed to clarify the relevance of this odd observation.

### Glutamatergic synaptic transmission at hippocampal SC–CA3>CA1 synapses is impaired in peradolescent *Itm2b*^*D*^ rats

**Figure 5:**
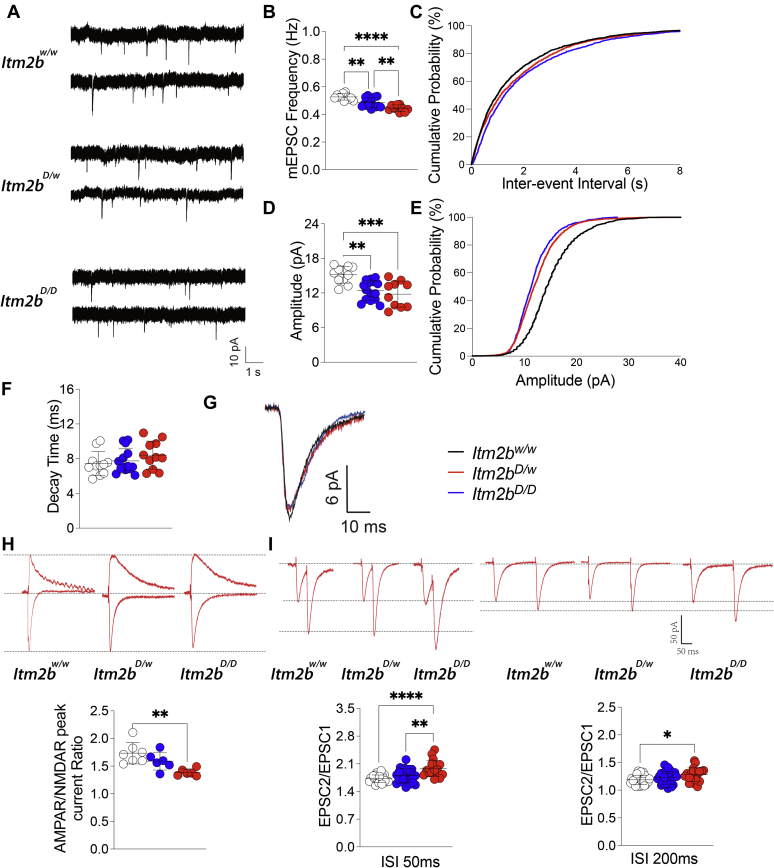
**Glutamatergic synaptic transmission is reduced at hippocampal SC–CA3>CA1 synapses of *Itm2b***^***D***^**KI rats.** Data are represented as mean ± SD and were analyzed by ordinary one-way ANOVA followed by post hoc Tukey's multiple comparisons test when ANOVA showed significant differences. We used the following animals: *Itm2b*^*w/w*^ N = 11 (4M/6, 3F/5, indicating that six recordings were obtained from the four males and five recordings from the three females), *Itm2b*^*D/w*^ N = 15 (3M/7, 4F/8), *Itm2b*^*D/D*^ N = 10 (3M/5, 3F/5). *A*, representative recording traces of mEPSC at SC–CA3>CA1 synapses. *B*, the Danish mutation causes a significant decrease in mEPSC frequency (ANOVA summary, F_(2, 33)_ = 20.66, *p* < 0.0001∗∗∗∗; post hoc Tukey's multiple comparisons test: *Itm2b*^*w/w*^*versus Itm2b*^*D/w*^, *p* = 0.003∗∗, *Itm2b*^*w/w*^*versus Itm2b*^*D/D*^, *p* < 0.0001∗∗∗∗; *Itm2b*^*D/w*^*versus Itm2b*^*D/D*^, *p* = 0.0051∗∗). *C*, cumulative probability of AMPAR-mediated mEPSC frequency interevent intervals. *D*, the Danish mutation causes a significant decrease in mEPSC amplitude (ANOVA summary, F_(2, 33)_ = 10.78, *p* = 0.0002∗∗∗; post hoc Tukey's multiple comparisons test: *Itm2b*^*w/w*^*versus Itm2b*^*D/w*^, *p* = 0.0016∗∗; *Itm2b*^*w/w*^*versus Itm2b*^*D/D*^, *p* = 0.0005∗∗∗; *Itm2b*^*D/w*^*versus Itm2b*^*D/D*^, *p* = 0.6691). *E*, cumulative probability of AMPAR-mediated mEPSC amplitude. *F*, in contrast, decay time of mEPSC was not significantly changed (ANOVA summary, F_(2, 33)_ = 1.292, *p* = 0.2882). *G*, average mEPSC of *Itm2b*^*D/D*^, *Itm2b*^*D/w*^, and *Itm2b*^*w/w*^ rats. *H*, AMPAR/NMDAR peak current ratio is significantly decreased in *Itm2b*^*D/D*^ rats (ANOVA summary, F_(2, 16)_ = 8.417, *p* = 0.0032∗∗; post hoc Tukey's multiple comparisons test: *Itm2b*^*w/w*^*versus Itm2b*^*D/w*^, *p* = 0.2393; *Itm2b*^*w/w*^*versus Itm2b*^*D/D*^, *p* = 0.0023∗∗; *Itm2b*^*D/w*^*versus Itm2b*^*D/D*^, *p* = 0.0818). Representative traces are shown on top of the graph (traces are averaged from 20 sweeps). Animals used: *Itm2b*^*w/w*^ N = 7 (4M/4, 3F/3), *Itm2b*^*D/w*^ N = 6 (3M/3, 3F/3), and *Itm2b*^*D/D*^ N = 6 (3M/3, 3F/3). *I*, average PPF at 50 ms (*left panel*) and 200 ms (*right panel*) interstimulus interval (ISI) shows that PPF is increased in *Itm2b*^*D/D*^ rats ISI (50 ms ISI PPF ANOVA summary: F_(2, 60)_ = 11.89, *p* < 0.0001∗∗∗∗; post hoc Tukey's multiple comparisons test: *Itm2b*^*w/w*^*versus Itm2b*^*D/w*^, *p* = 0.2315; *Itm2b*^*w/w*^*versus Itm2b*^*D/D*^, *p* < 0.0001∗∗∗∗; *Itm2b*^*D/w*^*versus Itm2b*^*D/D*^, *p* = 0.0031∗∗. 200 ms ISI PPF ANOVA summary: F_(2, 64)_ = 3.802, *p* = 0.0275∗; post hoc Tukey's multiple comparisons test: *Itm2b*^*w/w*^*versus Itm2b*^*D/w*^, *p* = 0.4504; *Itm2b*^*w/w*^*versus Itm2b*^*D/D*^, *p* = 0.0205∗; *Itm2b*^*D/w*^*versus Itm2b*^*D/D*^, *p* = 0.2462). Representative traces are shown on top of the panels. *p* < 0.05∗; *p* < 0.01∗∗; *p* < 0.001∗∗∗; and *p* < 0.0001∗∗∗∗. Animals used: *Itm2b*^*w/w*^ N = 18 (4M/10, 3F/8), *Itm2b*^*D/w*^ N = 20 (3M/10, 4F/10), and *Itm2b*^*D/D*^ N = 17 (3M/8, 3F/9). AMPAR, α-amino-3-hydroxy-5-methyl-4-isoxazolepropionic acid receptor; mEPSC, miniature excitatory postsynaptic current; NMDAR, *N*-methyl-d-aspartic acid; PPF, paired-pulse facilitation.

Bri2 modulates glutamatergic synaptic transmission at both presynaptic and postsynaptic termini of Schaeffer-collateral (SC) pathway–CA3>CA1 synapses (46). This function is compromised in both adult *Itm2b*^*D*^ and *Itm2b*^*B*^ KI mice (41). Here, we analyzed glutamatergic transmission at SC–CA3>CA1 synapses in young periadolescent *Itm2b*^*D*^ male and female rats. First, we analyzed miniature excitatory postsynaptic currents (mEPSCs), the frequency of which is determined, in part, by the probability of release (P*r*) of glutamatergic synaptic vesicles release (47). Thus, mEPSC frequency is regulated mostly by presynaptic mechanisms. As shown in Figure 5, *A*–*C*, the Danish *Itm2b* mutation caused a significant reduction in the frequency of mEPSC: this reduction is gene dosage dependent (*Itm2b*^*w/w*^ *versus Itm2b*^*D/w*^, *p* = 0.003; *Itm2b*^*w/w*^ *versus Itm2b*^*D/D*^, *p* < 0.0001; and *Itm2b*^*D/w*^ *versus Itm2b*^*D/D*^, *p* = 0.0051) and suggests a decrease in P*r* of glutamatergic synaptic vesicles.

The amplitude of mEPSC is instead dependent on postsynaptic α-amino-3-hydroxy-5-methyl-4-isoxazolepropionic acid receptor (AMPAR) responses. AMPAR-mediated mEPSC responses amplitude was also significantly decreased in *Itm2b*^*D*^ rats (Fig. 5, *A*, *D*, *E*, and *G*). Also in this case, the reduction is gene dosage dependent (*Itm2b*^*w/w*^ *versus Itm2b*^*D/w*^, *p* = 0.0016; *Itm2b*^*w/w*^ *versus Itm2b*^*D/D*^, *p* = 0.0005). Decay time of mEPSC was not significantly affected in *Itm2b*^*D*^ rats compared with littermate controls (Fig. 5, *A*, *F*, and *G*).

Since mEPSC AMPAR-mediated responses are reduced in amplitude, we measured the AMPAR/*N*-methyl-d-aspartic acid (NMDAR) peak current ratio in evoked responses. Consistent with the hypothesis that the Danish *Itm2b* mutation impairs AMPAR-mediated responses, the AMPAR/NMDAR peak current ratio was reduced in Danish KI rats (Fig. 5*H*). This difference was statistically different only between *Itm2b*^*w/w*^ and *Itm2b*^*D/D*^ rats, with *Itm2b*^*D/w*^ rats showing an intermedia phenotype.

Finally, we examined the effect of the pathogenic Danish mutation on paired-pulse facilitation (PPF). PPF is a form of short-term synaptic plasticity that is in part determined by changes in P*r* of glutamatergic synaptic vesicles (41, 43, 47). Facilitation at both 50 and 200 ms interstimulus interval (ISI) was significantly increased in *Itm2b*^*D/D*^ (Fig. 5*I*). Even in this case, the changes were gene dosage dependent (50 ms ISI: *Itm2b*^*w/w*^ *versus Itm2b*^*D/D*^, *p* < 0.0001; *Itm2b*^*D/w*^ *versus Itm2b*^*D/D*^, *p* = 0.0031; 200 ms ISI: *Itm2b*^*w/w*^ *versus Itm2b*^*D/D*^, *p* = 0.0205). Interestingly, also an increase in PPF is consistent with a decrease in P*r*, just like a decrease in mEPSC frequency. Overall, our data indicate that the pathogenic Danish *Itm2b* mutation alters glutamatergic synaptic transmission at excitatory hippocampal SC–CA3>CA1 synapses in periadolescent KI rats. These alterations are like those seen in *Itm2b* KO and *Itm2b*^*D*^/*Itm2b*^*B*^ KI adult mice.

## Discussion

The choice of the genetic approach and the model organisms used to model human diseases have major implications on the phenotypic expression of disease-associated genetic mutations. For the last 13 years, our laboratory has modeled AD and AD-like neurodegenerative disorders in mice, using a KI approach (3, 48, 49, 50, 51). The KI approach was preferred because it generates models genetically faithful to human diseases and makes no preconceived assumption about pathogenic mechanisms (except the unbiased genetic one). We have recently extended our KI modeling of familial and sporadic forms of AD and AD-related disorders to rats (38, 42, 43, 52, 53) because the rat is better suited for behavioral tests and other procedures that are important when studying neurodegenerative diseases. In addition, gene expression differences suggest that rats may be advantageous model of neurodegenerative diseases over mice. Alternative spicing of *Mapt* (12, 13, 14, 15), which forms NFTs and is mutated in frontotemporal dementia (16, 17, 18, 19, 20, 21, 22, 23), leads to expression of tau isoforms with three or four microtubule-binding domains (3R and 4R, respectively). Adult human and rat brains express both 3R and 4R tau isoforms (24): in contrast, adult mouse brains express only 4R tau (25), suggesting that the rat may be a better model organism for dementias with tauopathy.

To explore early dysfunctions that may underlie initial mechanisms leading to dementia, we studied young KI rats carrying the *Itm2b*^*D*^ FDD mutation. Consistent with the findings in *Itm2b*^*D/D*^ mouse KIs (41), we found that Bri2-ADan maturation is altered and accumulates in *Itm2b*^*D/D*^ primary neurons (Fig. 2). Analysis of APP metabolism in periadolescent *Itm2b*^*D*^ KI rats (Figs. 3 and 4) only showed subtle but significant changes in Aβ42 and Aβ43 steady-state levels, which were slightly increased and decreased, respectively (Fig. 3).

We have previously shown that the Danish and British *ITM2b* mutations lead to reduced glutamatergic neurotransmitter release and AMPAR-mediated responses in adult *Itm2b*^*B*^ and *Itm2b*^*D*^ mice. These reductions are like those seen in adult *Itm2b* KO mice (41, 46). Interestingly, we detected identical, gene dosage–dependent, presynaptic and postsynaptic glutamatergic transmission changes in the SC pathway of periadolescent *Itm2b*^*D*^ rats (Fig. 5). More specifically, the frequency of mEPSC and PPF is significantly decreased and increased, respectively, in *Itm2b*^*D*^ rats, suggesting a presynaptic reduction of the P*r* of glutamatergic synaptic vesicles. In addition, mEPSC amplitude and the AMPAR/NMDAR peak current ratio were both decreased in *Itm2b*^*D*^ rats, suggesting a postsynaptic reduction of AMPAR-mediated responses. Collectively, these data together with our previously published observations indicate that the synaptic transmission alteration caused by Danish mutation occurs early in life and are neither species nor gene-editing technology specific. These studies underlie the potential relevance of our studies to functional changes caused by the pathogenic *ITM2b* mutations in humans.

Given the functional and pathological interaction between APP and BRI2 (26, 27, 28, 29, 30, 31, 32, 33, 34, 35, 36), it is possible that the presence of human Aβ in the rat model may lead to an earlier manifestation of synaptic plasticity deficit in rat as compared with mice, which express rodent Aβ. Moreover, the evidence that both APP and BRI2 tune excitatory synaptic transmission, and that these functions are altered by pathogenic mutations in both APP and BRI2 (37, 41, 46, 54, 55, 56), suggests that early alterations in glutamatergic transmission may underlie initial pathogenic mechanisms in dementia. Future studies will be needed to test these hypotheses.

## Experimental procedures

### Rats and ethics statement

Rats were handled according to the National Institutes of Health Ethical Guidelines for Treatment of Laboratory Animals. The procedures were described and approved by the Institutional Animal Care and Use Committee at Rutgers (Institutional Animal Care and Use Committee; protocol number: PROTO201702513).

### Generation of rats expressing the Danish Itm2b mutation (Itm2^D^ rats)

The rat *Itm2b* gene (GenBank accession number: NM_001006963.1; Ensembl: ENSRNOG00000016271) is located on rat chromosome 15. It comprises six exons, with ATP start codon in exon 1 and TGA stop codon in exon 6. The FDD mutation (TTTAATTTGTTCTTGAACAGTCAAGAAAAACATTAT) KI site in oligo donor was introduced into exon 6, which is the target site by homology-directed repair. A silent mutation (GTG to GTC) was also introduced to prevent the binding and recutting of the sequence by Cas9 after homology-directed repair. The detailed procedures are reported in the supporting information file.

### Standard RNA-Seq analysis

Total brain RNA from 21-day-old *Itm2b*^*D/D*^ and *Itm2b*^*w*/*w*^ rats (two male and two females per each genotype) was extracted with RNeasy RNA Isolation kit (Qiagen). Standard RNA-Seq procedures and data analysis were performed by Genewiz following proprietary methods (https://cdn2.hubspot.net/hubfs/3478602/NGS/RNA-Seq/GENEWIZ_RNA-Seq_Technical_Specifications_US.pdf). Student's *t* test was used for all analyses, with data presented as mean ± SD.

### Protein preparation, Western blots, and ELISA of rat brain

These procedures were performed as previously described (42, 53). Briefly, rats were anesthetized with isoflurane and perfused *via* intracardiac catheterization with ice-cold PBS. Brains were extracted and homogenized with a glass-teflon homogenizer in 250 mM sucrose, 20 mM Tris base, pH 7.4, 1 mM EDTA, 1 mM EGTA plus protease and phosphatase inhibitors (Thermo Fisher Scientific). All steps were carried out on ice. Homogenates were solubilized with 1% NP-40 for 30 min rotating and spun at 20,000*g* for 10 min. Supernatants were collected, and protein content was quantified by Bradford.

For Western blot analyses, proteins were diluted with PBS and LDS sample buffer—10% β-mercaptoethanol (Invitrogen; NP0007) and 4.5 M urea to 1 μg/μl, loaded on a 4 to 12% Bis–Tris polyacrylamide gel (Bio-Rad; 3450125), and transferred onto nitrocellulose at 25 V for 7 min using the Trans-blot Turbo system (Bio-Rad). Blotting efficiency was visualized by red Ponceau staining on membranes. For dot-blot analysis, 2.5 μg of material was directly spotted with a p20 pipette on a nitrocellulose membrane. Dot membrane was also visualized by red Ponceau after it was totally dried. Membranes were blocked in 5% milk (Bio-Rad; 1706404) for 30 min and washed in PBS/Tween-20 to 0.05%. Primary antibodies were applied dilution in blocking solution (Thermo; 37573). The following antibodies were used: polyclonal anti-Bri2 serum test bleeds provided by Cell Signaling Technology was used at 1:500 with overnight shaking at 4 °C. APP-Y188 (Abcam; 32136), Oligomer Aβ A11 (shared by Rakez Kayed's laboratory), LC3A (CST; 4599), and LC3B (CST; 2775) were used at 1:1000 with same other condition. Secondary antibodies (either antimouse [Southern Biotech; 1031-05] or a 1:1 mix of anti-rabbit [Southern Biotech; OB405005] and anti-rabbit [Cell Signaling; 7074]) were diluted 1:1000 in 5% milk and used against either mouse or rabbit primary antibodies for 1 h at room temperature, with shaking. Membranes were washed with PBS/Tween-20 to 0.05% (three times, 10 min each time), developed with West Dura ECL reagent (Thermo: PI34076) and visualized on a ChemiDoc MP Imaging System (Bio-Rad). Signal intensity was quantified with Image Lab software (Bio-Rad). Data were analyzed using Prism software (GraphPad Software, Inc) and represented as mean ± SD.

For analysis of human Aβ peptides and sAPPα/sAPPβ, brain lysates were diluted at 4 μg/μl. Aβ38, Aβ40, and Aβ42 were measured with V-PLEX Plus Aβ Peptide Panel 1 6E10 (K15200G; Meso Scale Discovery), and sAPPα/sAPPβ were measured with sAPPα/sAPPβ (K15120E; Meso Scale Discovery). Plates were read on a MESO QuickPlex SQ 120. Aβ43 was quantified using the IBL human Aβ43 Assay Kit #27710.

### Primary hippocampal neuron culture

Rat hippocampal neurons were prepared from *Itm2b*^*w/w*^ and *Itm2b*^*D/D*^ postnatal day 1 pups. Briefly, after removal of meninges, the hippocampi were collected in Hank's balanced salt solution without magnesium and calcium, 1 mM sodium pyruvate, 0.1% glucose, and 10 mM Hepes. Hippocampi were dissected into single cell by trituration followed by 15-min incubation at 37 °C in 0.25% trypsin. Cells were subsequently treated with 0.1% DNAse (Sigma; dn25) in plating media (beta-mercaptoethanol, 10% fetal bovine serum, 0.09% glucose, 1 mM sodium pyruvate, 2 mM glutamine, and 1× penicillin/streptomycin). Cells were filtered through a Falcon 70 μm nylon cell strainer and were plated in poly-l-lysine–pretreated 12-well plate (300,000 cells/well) in neurobasal media, 1× B-27, 2 mM glutamine, and 1× penicillin/streptomycin. Half of the culture media was changed every 2 days. The six *Itm2b*^*w/w*^ and *Itm2b*^*D/D*^ cultures shown in Figure 2 were derived from one P1 pup. For each animal, equal amounts of cells were cultured into two wells. For each biological replicate, one well was treated with PBS and one well with chloroquine.

### Pharmacological treatment and sample preparation

After 9 days in culture, primary neurons were treated with 50 μM chloroquine (Cell Signaling; 14774s) or PBS (Veh) for 18 h. After treatment, cells were washed with PBS and lysed in radioimmunoprecipitation buffer with protease/phosphatase inhibitor for 15 min on ice. Lysed cells were centrifuged at full speed for 15 min. Cell lysates were quantified and analyzed by Western blot as described earlier for brain lysates.

### Electrophysiological recording

These procedures were performed as previously described (52). Briefly, rats were anesthetized with isoflurane and perfused intracardially with an ice-cold cutting solution containing (in millimolar) 120 choline chloride, 2.6 KCl, 26 NaHCO_3_, 1.25 NaH_2_PO_4_, 0.5 CaCl_2_, 7 MgCl_2_, 1.3 ascorbic acid, and 15 glucose, prebubbled with 95% O_2_/5% CO_2_ for 15 min. The brains were rapidly removed from the skull, and coronal brain slices containing the hippocampal formation (350 μm thick) were prepared in the ice-cold cutting solution bubbled with 95% O_2_/5% CO_2_ using Vibratome VT1200S (Leica Microsystems) and then incubated in an interface chamber in artificial cerebrospinal fluid (ACSF) containing (in millimolar): 126 NaCl, 3 KCl, 1.2 NaH_2_PO_4_; 1.3 MgCl_2_, 2.4 CaCl_2_, 26 NaHCO_3_, and 10 glucose (at pH 7.3), bubbled with 95% O_2_ and 5% CO_2_ at 30 °C for 1 h and then kept at room temperature. The hemislices were transferred to a recording chamber perfused with ACSF at a flow rate of ∼2 ml/min using a peristaltic pump. Experiments were performed at 28.0 °C ± 0.1 deg. C.

Whole-cell recordings in the voltage-clamp mode (−70 mV) were made with patch pipettes containing (in millimolar): 132.5 Cs gluconate, 17.5 CsCl, 2 MgCl_2_, 0.5 EGTA, 10 Hepes, 4 ATP, and 5 QX-314, with pH adjusted to 7.3 by CsOH. Patch pipettes (resistance, 8–10 MΩ) were pulled from 1.5-mm thin-walled borosilicate glass (Sutter Instruments) on a horizontal puller (model P-97; Sutter Instruments). Basal synaptic responses were evoked at 0.05 Hz by electrical stimulation of the SC afferents using concentric bipolar electrodes. CA1 neurons were viewed under upright microscopy (FN-1; Nikon Instruments) and recorded with Axopatch-700B amplifier (Molecular Devices). Data were low-pass filtered at 2 kHz and acquired at 5 to 10 kHz. The series resistance (Rs) was consistently monitored during recording in case of reseal of ruptured membrane. Cells with Rs >20 MΩ or with Rs deviated by >20% from initial values were excluded from analysis. EPSCs were recorded in ACSF containing the gamma aminobutyric acid-A receptors inhibitor bicuculline methiodide (15 μM). The stimulation intensity was adjusted to evoke EPSCs that were 40% of the maximal evoked amplitudes (“test intensity”). About 5 to 10 min after membrane rupture, EPSCs were recorded for 7 min at a test stimulation intensity that produced currents of ∼40% maximum. For recording of paired-pulse ratio, paired-pulse stimuli with 50 or 200 ms interpulse interval were given. The paired-pulse ratio was calculated as the ratio of the second EPSC amplitude to the first. For recording of AMPAR/NMDAR peak current ratio, the membrane potential was first held at −70 mV to record only AMPAR current for 20 sweeps with 20-s intervals. Then the membrane potential was turned to +40 mV to record NMDAR current for 20 sweeps with perfusion of 5 μM NBQX to block AMPAR. Mini EPSCs were recorded by maintaining neurons at −70 mV with ACSF containing action potential blocker (1 μM tetrodotoxin) and GABA-A receptor inhibitors (15 μM bicuculline methiodide). mEPSCs were recorded for ∼10 min. Data were collected with Axopatch 700B amplifiers and analyzed with pCLAMP10 software (Molecular Devices). mEPSCs are analyzed using Mini Analysis Program.

### Statistics

All the experiments mentioned in the article were analyzed by one-way ANOVA or two-way ANOVA as indicated. Data showing statistical significance by one-way ANOVA or two-way ANOVA were subsequently analyzed by either Tukey's multiple comparisons test or Sidak's multiple comparisons. All statistical analyses were performed using Prism 9 software.

## Data availability

The datasets used and/or analyzed during the current study are available from the corresponding author on reasonable request.

## Supporting information

This article contains supporting information.

## Conflict of interest

The authors declare that they have no conflicts of interest with the contents of this article.
