# Supplementary material for: A familial Danish dementia rat shows impaired presynaptic and postsynaptic glutamatergic transmission
Source: J Biol Chem. 2021 Aug 18;297(3):101089. doi: 10.1016/j.jbc.2021.101089 (PMC8429969; doi:10.1016/j.jbc.2021.101089)
Supplement: Supplemental Figures S1–S3 and Table S1 [file mmc1.pdf]

▪ **A Familial Danish dementia rat shows impaired pre- and postsynaptic glutamatergic transmission**

Tao Yin[1], Wen Yao[1,2], Kelly A. Norris[1] & Luciano D'Adamio[1]

Supporting Information

1. Extended Experimental Procedures.
2. Figure S1 – Western blot of total rat brain lysates with the anti-Bri2 antibody.
3. Figure S2 – Western blot analysis of Bri2 expression in primary hippocampal neurons treated with 50µM chloroquine for 18h. In this experiment we used a Rabbit IgG monoclonal antibody that is being developed by Cell signaling technology (supporting data shown in Figure 2A of the manuscript).
4. Figure S3 – Analysis of AMPAR/NMDAR AUC (Area Under Curve) Ratio and decays of the NMDAR currents (supporting data shown in Figure 5H of the manuscript).
5 Table S1, which reports the detailed statistical analysis results for Figure S2.

1. Extended Experimental procedures

The KI founder F0-*Itm2b*^{D} rat was generated by CRISPR/Cas-mediated genome engineering. The rat *Itm2b* gene (GenBank accession number: NM_001006963.1; Ensembl: ENSRNOG00000016271) is located on rat chromosome 15. It comprises 6 exons, with ATG start codon in exon 1 and TGA stop codon in exon 6. The Danish mutation sequence was introduced in exon 6 to replace the stop codon (with targeting sequence, flanked by 140 bp homologous sequences combined on both sides). A silent mutation (GTG to GTC, Figure S1) was introduced to prevent the binding and re-cutting of the sequence by Cas9 after homology-directed repair. One pair of gRNA targeting vector and oligo donor were designed as shown in Figure S1.

Cas9 mRNA, gRNA generated by *in vitro* transcription and oligo donor were co-injected into fertilized eggs for KI rat production. To verify CRISPR-induced mutation, the pups were genotyped by PCR, followed by sequence analysis. The rat *Itm2b* locus was amplified by PCR with the following specific forward (F) and reverse (R) primers: F-AATGTGGAAATTATGGGGTGGAT, R-GATGAAGAGACAGTGAAGCCCTG.

Cas9 RNA, sgRNA and oligo donor are co-injected into zygotes, but homology-directed repair can occur even after few cell cycles. Thus, injected rats can have a mixture of correctly targeted alleles and alleles carrying aberrant mutations or no mutations. To identify rats carrying correctly targeted *Itm2b* alleles, the PCR products were cloned into TA vectors and were sequenced using forward primer F-AGTGACATGCTTCTTTATGTCT. Rat-ID#83 was identified as a positive chimeric founder (F0-*Itm2b*^{D} rat).

Figure S1. Oligo donor sequence, with the FDD mutated nucleotide in red and the silent TCC to TCG mutation in red and yellow highlight. Sequences of gRNA1 and gRNA2 are shown.

Oligo donor

GCGTGAAGCCAGTAACTGTTTCACCATTCGGCATTTTGAGAACAAATTTGCTGTCGAAACTTT[illegible]
ATTTGTTTTAATTTGTTCTTGAACAGTCAAGAAAAACATTATTGAGACGTCAAGAAAAAAAGTG[illegible]
GGAGAAATTCAATGCCACAGCATACCCTGCCCCTTTGTATTTTGTGC

Pair

gRNA1: TTTTGAGAACAAATTTGCTGTGG

gRNA2: AGACGTCAAGAAAAAAAGTGAGG

The links of gRNA on VectorBuilder:

gRNA1: http://www.vectorbuilder.com/design/report/de681835-f2aa-4df1-8511-36e18ccbdb2f

gRNA2: http://www.vectorbuilder.com/design/report/27323bb6-2b38-4da4-857f-1a61ba06dbb2

Off-target analysis of targeting sequence gRNA1**:** GAAAATGAGCCCGAAGGTGATGG**.** We identified five potential off-target sites for gRNA1. F0-*Itm2b*^{D} rat was analyzed for mutations in these most likely off-target mutation sites. Mismatched bases are in red. These sites have been amplified by PCR and sequenced.

Potential off-target sites: (The upper panel is the targeting sequence, and the down panel is the potential off-target sites sequence, the mismatches base between them is show in red)

119730654-ACTTGAGAAAAAATTTGCTGCAG-119730676: **Chr X** potential off-target sites sequence
TTTTGAGAACAAATTTGCTGTGG: Targeting sequence
A 289bp PCR product was generated using primers F-GTCACAGTTTGACAGTCATGGTCAAG and R-GTCAGAAATGGGAGAAGTCAAGAAAT, and sequenced using the F-primer showed no mutations in F0-*Itm2b*^{D} rat.

17934473-TCTTGAGAAGAAATTTGCTGGAG-17934495: **Chr14** potential off-target sites sequence:
TTTTGAGAACAAATTTGCTGTGG: Targeting sequence
A 390bp PCR product was generated using primers F-GTTCATGGAATCCTGCCTAAAGTTG and R-GAATATGTGGAATGTCACTAAATCACAAC, and sequenced using the F-primer showed no mutations in F0-*Itm2b*^{D} rat.

25587824-TTTTGATAACAAATTTGCAGAAG-25587846: **Chr 4** potential off-target sites sequence:
TTTTGAGAACAAATTTGCTGTGG: Targeting sequence
A 397bp PCR product was generated using primers F-GGCTATGGGATTCAAGACCCTTTG and R-CTCCTGTTTCTATGTTAATCACATCGG, and sequenced using the F-primer showed no mutations in F0-*Itm2b*^{D} rat.

46760517-TGTTAAAAACAAATTTGCTGTAG-46760539: **Chr 1** potential off-target sites sequence:
TTTTGAGAACAAATTTGCTGTGG: Targeting sequence
A 341bp PCR product was generated using primers F-CAAGAGAAGCTGAAAATTCCACCTC and R-CTCCCTCGTCTCCACTCTTCTGC, and sequenced using the F-primer showed no mutations in F0-*Itm2b*^{D} rat.

62703744-TGTTGAGTACCAATTTGCTGGGG-62703766: **Chr 14** potential off-target sites sequence:
TTTTGAGAACAAATTTGCTGTGG: Targeting sequence
A 484bp PCR product was generated using primers F-AACCCTCTATGATGTACTTTTCACATTC and R GTAGTTTAGCTGGTTTCTAGTATGAGGC, and sequenced using the F-primer showed no mutations in F0-*Itm2b*^{D} rat.

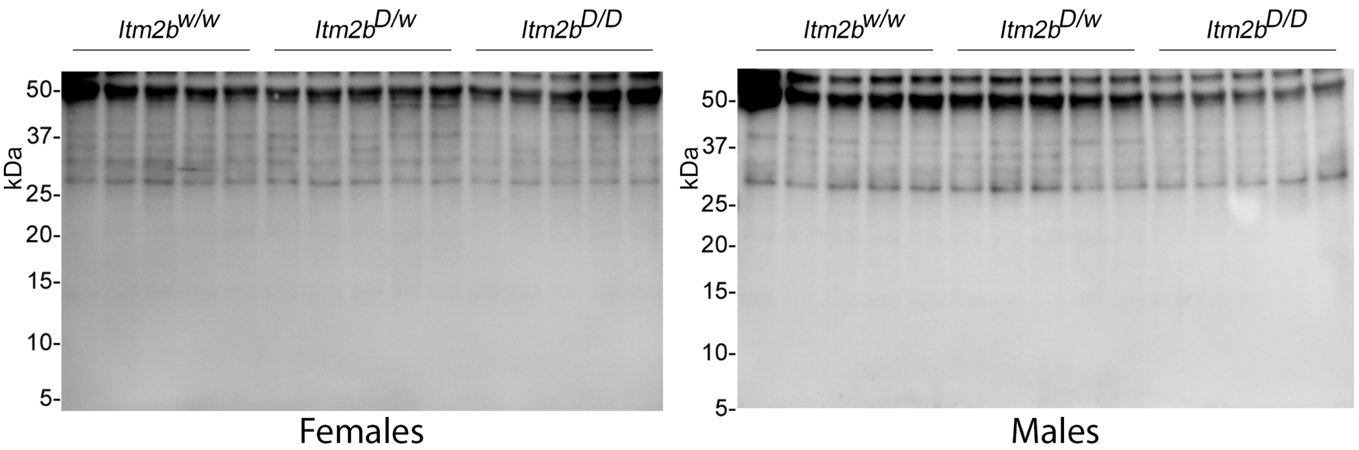

Figure S1 – Western blot of total brain lysates isolated from 8 weeks old *Itm2b*$^{D/D}$, *Itm2b*$^{D/w}$ and *Itm2b*$^{w/w}$ rats. We used 5 female and 5 male rats per genotype. There are too many non-specific bands, which makes difficult to rigorously assess Bri2 expression in rat brains.

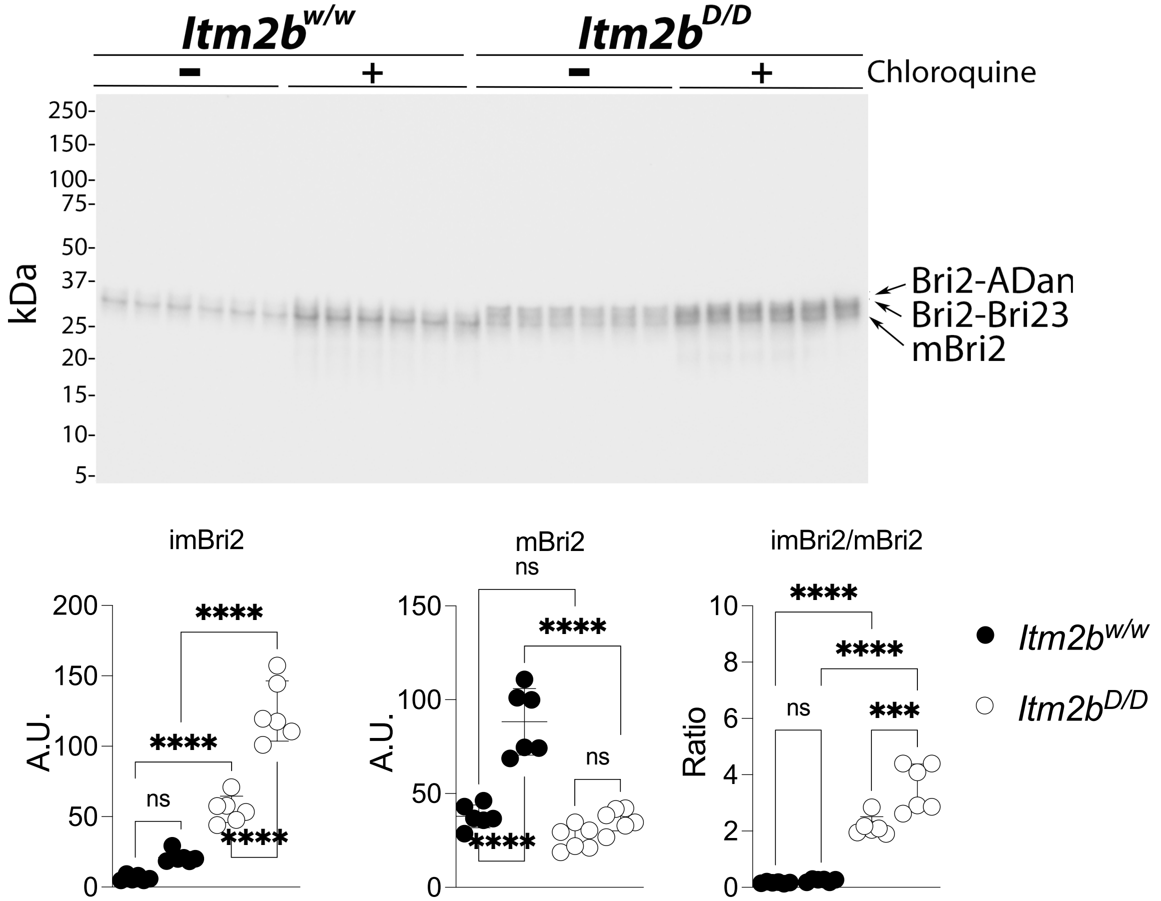

Figure S2 – WB analysis of Bri2 from primary hippocampal neurons isolated from *Itm2b*$^{w/w}$ and *Itm2b*$^{D/D}$ P1 pups treated with (+) or without (-) 50µM chloroquine for 18h. Primary antibody: purified Rabbit monoclonal antibody produced by Cell signaling technology (Clone 10G1). Quantification of Bri2 levels. Data are represented as mean ± SD and analyzed by ordinary two-way ANOVA followed by post-hoc Sidak's multiple comparisons test when ANOVA showed significant differences. The detailed statistical analysis results are shown in Table S1.

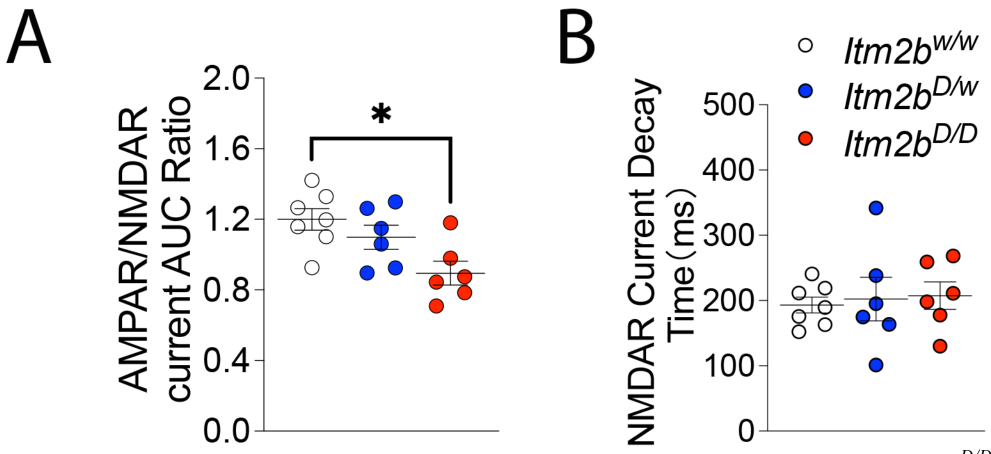

Figure S3 – (A) AMPAR/NMDAR AUC Ratio is significantly decreased in *Itm2b*$^{D/D}$ rats [ANOVA summary, F (2, 16) = 5.613, P=0.0142*; post-hoc Tukey's multiple comparisons test: *Itm2b*$^{w/w}$ vs *Itm2b*$^{D/w}$, P=0.5264; *Itm2b*$^{w/w}$ vs *Itm2b*$^{D/D}$, P=0.0115*; *Itm2b*$^{D/w}$ vs *Itm2b*$^{D/D}$, P=0.1145]. Meanwhile, the decay time of NMDAR current were not significantly changed [ANOVA summary, F (2, 16) = 0.1050, P=0.9010]. Representative traces are shown in Figure 5H.

	Ordinary two-way ANOVA		
imBri2	Source of Variation	F (DFn, DFd)	P
	Interaction	F (1, 20) = 31.98	P<0.0001
	treatment	F (1, 20) = 75.66	P<0.0001
	genotype	F (1, 20) = 246.1	P<0.0001
	post-hoc Sidak's multiple comparisons test	Summary	Adjusted P
	$Itm2b^{w/w}$(Veh) vs. $Itm2b^{D/D}$(Veh)	****	<0.0001
	$Itm2b^{w/w}$(Veh) vs. $Itm2b^{w/w}$(Chlo)	ns	0.2356
	$Itm2b^{D/D}$(Veh) vs. $Itm2b^{D/D}$(Chlo)	****	<0.0001
	$Itm2b^{w/w}$(Chlo) vs. $Itm2b^{D/D}$(Chlo)	****	<0.0001
mBri2	Source of Variation	F (DFn, DFd)	P
	Interaction	F (1, 20) = 22.82	P=0.0001
	treatment	F (1, 20) = 51.26	P<0.0001
	genotype	F (1, 20) = 57.32	P<0.0001
	post-hoc Sidak's multiple comparisons test	Summary	Adjusted P
	$Itm2b^{w/w}$(Veh) vs. $Itm2b^{D/D}$(Veh)	ns	0.3196
	$Itm2b^{w/w}$(Veh) vs. $Itm2b^{w/w}$(Chlo)	****	<0.0001
	$Itm2b^{D/D}$(Veh) vs. $Itm2b^{D/D}$(Chlo)	ns	0.4948
	$Itm2b^{w/w}$(Chlo) vs. $Itm2b^{D/D}$(Chlo)	****	<0.0001
imBri2/mBri2	Source of Variation	F (DFn, DFd)	P
	Interaction	F (1, 20) = 12.38	P=0.0022
	treatment	F (1, 20) = 15.59	P=0.0008
	genotype	F (1, 20) = 206.0	P<0.0001
	post-hoc Sidak's multiple comparisons test	Summary	Adjusted P
	$Itm2b^{w/w}$(Veh) vs. $Itm2b^{D/D}$(Veh)	****	<0.0001
	$Itm2b^{w/w}$(Veh) vs. $Itm2b^{w/w}$(Chlo)	ns	0.9998
	$Itm2b^{D/D}$(Veh) vs. $Itm2b^{D/D}$(Chlo)	***	0.0002
	$Itm2b^{w/w}$(Chlo) vs. $Itm2b^{D/D}$(Chlo)	****	<0.0001

Table S1. Statistical analysis results for data shown in Figure S2.
